# An Electrically Tunable Dual-Wavelength Refractive Index Sensor Based on a Metagrating Structure Integrating Epsilon-Near-Zero Materials

**DOI:** 10.3390/s20082301

**Published:** 2020-04-17

**Authors:** Zhenya Meng, Hailin Cao, Run Liu, Xiaodong Wu

**Affiliations:** 1 School of Microelectronics and Communication Engineering, Chongqing University, Chongqing 400044, China; 2 State Key Laboratory of Power Transmission Equipment & System Security and New Technology, Chongqing University, Chongqing 400044, China

**Keywords:** refractive index sensor, narrowband perfect absorber, epsilon-near-zero materials, metagrating

## Abstract

In this paper, a reconfigurable sensing platform based on an asymmetrical metal-insulator-metal stacked structure integrating an indium tin oxide (ITO) ultrathin film is proposed and investigated numerically. The epsilon-near-zero (ENZ) mode and antisymmetric mode can be resonantly excited, generating near-perfect absorption of over 99.7% at 1144 and 1404 nm, respectively. The absorptivity for the ENZ mode can be modulated from 90.2% to 98.0% by varying the ENZ wavelength of ITO by applying different voltages. To obtain a highly sensitive biosensor, we show that the proposed structure has a full-width at half-maximum (FWHM) of 8.65 nm and a figure-of-merit (FOM) of 24.7 with a sensitivity of 213.3 nm/RI (refractive index) for the glucose solution. Our proposed device has potential for developing tunable biosensors for real-time health monitoring.

## 1. Introduction

Refractive index (RI) sensors, as bio-optical sensors that can detect tiny RI changes, have attracted considerable research interest due to their extensive application in biological and chemical sensing, including pH value measurement, detection of the solution concentration, environmental monitoring, and molecular structure determination [1,2,3,4,5,6,7]. With RI sensors, the label-free detection of molecule concentrations depends on the detection of variation in the refractive index, which does not require the sample to be marked with fluorescent dyes due to bonding events. The detection ability in optical biosensors can be described by two main parameters: the sensitivity and full-width at half-maximum (FWHM) of the absorptivity [8,9]. The figure of merit (FOM) of RI sensing can be defined by combining sensitivity S (the wavelength shift per RI unit) and FWHM as $FOM=\frac{S}{FWHM},$ where S=Δλ/Δn, and Δλ and Δn represent variation of the RI and the shifting wavelength at the absorption peak, respectively. Therefore, high-precision RI sensors should have both a high sensitivity and a narrow FWHM. Many RI sensors have been designed and fabricated based on optical fibers, surface plasmon resonances, and varying microcavities [10,11,12,13,14,15,16,17,18,19]. For instance, an RI sensor using a core micro-structured optical fiber was proposed by Li et al., who achieved a detection limit of $6.02\times 10^{-6}$ RI units with the RI ranging from 1.3320 to 1.3465 RI units. A gas RI sensor with a high sensitivity applying a hollow-core photonic bandgap fiber and the Fabry–Perot interferometer was experimentally demonstrated [20,21]. Metamaterial perfect absorbers (MMPAs) are also appropriate candidates for RI sensors because metamaterials with nanostructures can be used to optionally control the behavior of light at the nanoscale, such as the reflection, transmission, absorption, and enhancement of light [22,23,24,25].

MMPAs mainly benefit from metal or dielectric ohmic loss for applications in the fields of photonic modulators, thermal emitters, optical filters, solar energy harvesting, microbolometers, and sensors [26,27,28,29,30,31,32]. Since the first demonstration of MMPAs by Landy et al. at a narrow microwave frequency band using an electric ring resonator in 2008, the majority of theoretical and experimental work on MMPAs has been conducted in the microwave to optical spectral range [22,33]. Over the past decade, perfect light absorption has attracted much attention due to the wide applications in solar energy, detection, and sensing [34,35,36]. The absorbers can be classified into broadband absorbers and narrowband absorbers, according to their bandwidth. Broadband MMPAs can be implemented by creating contiguous multiple resonances or designing stacked multilayer structures in the vertical direction [37,38,39]. Uniform absorption in the radio and optical spectral range has extensive applications in electromagnetic shielding and thermophotovoltaics. However, some applications, such as optical sensing and modulation, need absorbers with an ultra-narrow spectral range for light absorption [2,40,41]. Therefore, narrowband MMPAs are important for the design of optical biosensing.

Currently, most narrowband MMPAs are based on two kinds of structures: the metamaterial (MM) resonator structure or the metal-dielectric-metal (MDM) structure [42,43,44,45]. For example, Lu et al. presented a metal (nanobar array with nanoslits)-dielectric-metal infrared absorber, which attained a full-width at half-maximum (FWHM), absorption, and figure-of-merit (FOM) of 8 nm, 95%, and 25, respectively [24]. Li et al. proposed a perfect absorber consisting of gold nanobars and a photonic microcavity in the infrared range with a narrow FWHM of 40.8 nm [46]. In [47], a maximum absorption of 95.4% and an FWHM value of 33 nm were achieved using a continuous type of metal-insulator-metal structure. Chen et al. theoretically designed and proved a dual-band perfect absorber composed of square-patch-based MDM structures via exciting the surface plasmon polariton (SPP) mode and Rayleigh anomaly, and the FWHM values for those two modes were 12 and 0.23 nm, respectively [8]. However, most of the absorbers did not simultaneously achieve near-perfect optical absorption with an extremely narrow bandwidth and multi-band. The electrical tunability achieved in electro-optical switching was not discussed in the above absorbers for RI sensors, which limits the applications of MMPAs in reconfigurable sensors.

**Figure 1:**
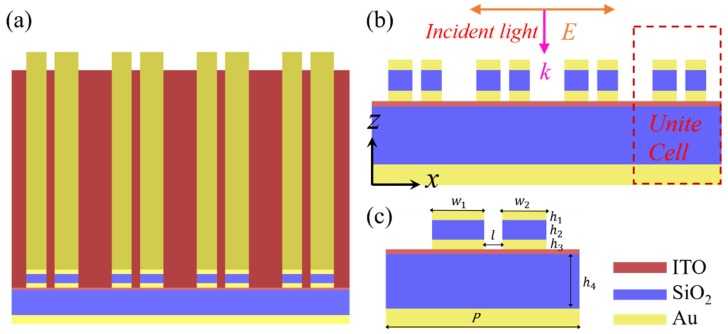
(**a**) Schematic of the metal-insulator-metal-insulator-metal (MIMIM) structure; (**b**) cross-section of the MIMIM structure and the incident light configuration; (**c**) cross-section of the MIMIM structure of a unit cell with dimension and material labels.

To overcome the dilemma, in this paper, an electrical tunable dual-band refractive index sensor is proposed and demonstrated numerically based on an asymmetric metagrating structure integrating an epsilon-near-zero (ENZ) ultrathin film, as shown in Figure 1. The designed structure can accomplish an extreme absorption of over 99.7% with an ultra-narrow FWHM of 8.65 nm. Based on [48,49,50,51,52], the ultrathin film of the ENZ materials that support bound ENZ modes can be used to achieve the perfect absorption of light. Generally, the thickness of the ENZ film should be less than $\lambda_{p}/50$. to excite the ENZ mode [53]. Here, a 6 nm indium tin oxide (ITO) film is taken as an ENZ material working in the ENZ regime to excite the ENZ mode. 

## 2. Materials, Structural Design, and Methods

Figure 1a depicts the geometrical configuration of the proposed structure consisting of an alternating metal-insulator-metal-insulator-metal (MIMIM) stacked array to produce ultra-narrow light absorption. The designed structure is composed of a periodic asymmetric gold (Au)-silicon dioxide (SiO_2_)-Au grating on the top and an Au ground layer at the bottom, separated by an SiO_2_ spacer layer deposited on the glass substrate. The three-layer nano-grating coupling the energy of incident light into the designed device is etched through the first three layers of the five-layer metal-insulator structure. $w_{1}$, $w_{2},$ and l represent the widths of the two stripes and the distance between the two stripes of asymmetric nano-grating, respectively (Figure 1c). The ITO ultra-thin film taken as an ENZ material is integrated into the device between the grating and SiO_2_ dielectric layer, which is able to excite the ENZ mode by coupling the energy of the incident light guiding via nano-grating. 

The optical properties of the designed device were numerically investigated based on the full-wave finite-difference time-domain (FDTD) algorithm. For an absorber, the sum of absorptivity (A), reflectivity (R), and transmissivity (T) is equal to 1. In this structure, the transmission of light is completely blocked by the gold mirror layer, which is optically thick. Consequently, the spectral absorption rate is only determined by reflection, A = 1 − T. We previously applied the three-dimensional (3D) Lumerical FDTD solution software to calculate the absorptivity, reflectivity, and characteristics of the optical field [54]. Nevertheless, the two-dimensional (2D) simulation model is applied here due to the symmetry of the proposed structure. Figure 1b shows a cross-section of the MIMIM structure and the incident light configuration in the simulation software. All simulations were executed under normal illumination of TM (Transverse Magnetic) polarized light (plane wave), polarized in the x-direction, with the periodic boundary condition of the unit cell set along the x- and y-direction. To eliminate scattering light, the perfectly matched layers were added along the z-axis direction.

The refractive indexes (RIs) of Au and SiO_2_ were taken from Palik [55]. The complex permittivity of ITO can be calculated via the following equation using the Drude model [56]:

(1) $$\varepsilon \left(\omega \right)=\varepsilon_{\infty }-\frac{\omega_{P}^{2}}{\omega \left(\omega +i\Gamma \right)},$$

where ω is the frequency and the optical constants $\omega_{P}$ and Γ represent the plasma frequency and collision rate of charge carriers, respectively. The plasma frequency $\omega_{P}$ can be obtained from the following equation:

(2) $$\omega_{P}^{2}=\frac{N_{0}e^{2}}{\varepsilon_{0}m^{*}},$$

where $N_{0}$ and $m^{*}$ represent the bulk free carrier concentration and the effective mass of electrons, respectively. Here, the electronic charge $e=1.6\times 10^{-19}\;C$, and the permittivity in free space $\varepsilon_{0}=$ 8.85 $\times 10^{-12}\;C^{2}/{\mathrm{Nm}}^{2}$. The real and imaginary permittivities of ITO were obtained by fitting the experimental data taken from [56]. As a result, the ENZ wavelength $\lambda_{ENZ}=1403\;\mathrm{nm},$ when Real (ε(ω))=0.

**Figure 2:**
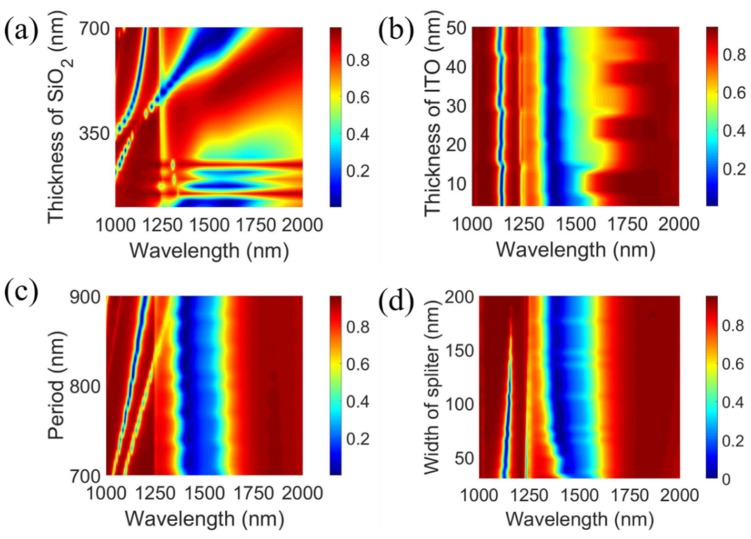
Reflectivity for the different thicknesses of (**a**) SiO_2_ and (**b**) indium tin oxide (ITO), (**c**) period, and (**d**) width of the splitter.

To produce a dual-wavelength biosensor with a high precision, the dimension of the proposed device should first be investigated based on FDTD simulation. The reflectivities for the different thicknesses of SiO_2_ and ITO, period, and width of the splitter are presented in Figure 2. In Figure 2a, it is easy to see that strong absorption is generated at wavelengths of 1090~1200 nm and 1250~1600 nm for SiO_2_ thicknesses ranging from 400 to 700 nm. There are several parameters that affect the absorption. Therefore, the rough range of dimension is given based on an analysis of parameter sweep, which is less than 28 nm for ITO, 750~900 nm for the period, and 30~150 nm for the splitter.

**Figure 3:**
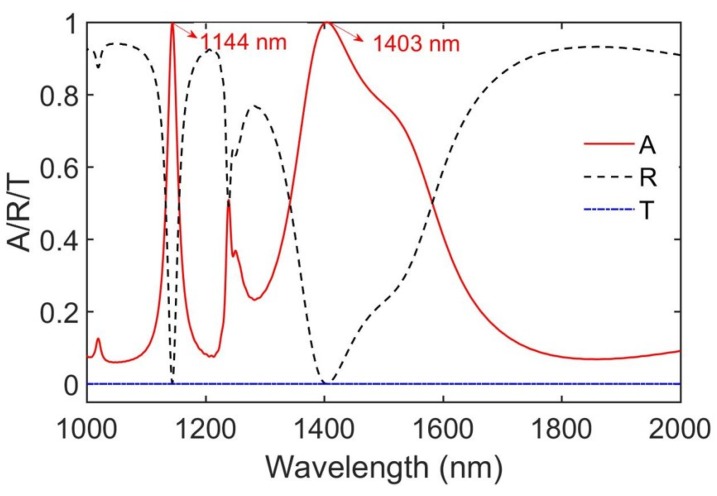
The absorptivity (A), reflectivity (R), and transmissivity (T) of the proposed structure under the normal incidence.

The simulated absorptivity, reflectivity, and transmissivity of the MIMIM structure integrating an ITO film are depicted in Figure 3 for the optimization parameters $h_{1}=h_{3}=100\;\mathrm{nm},\;h_{2}=200\;\mathrm{nm},\;h_{4}=603\;\mathrm{nm},\;\mathrm{and}\;l=$ 82.75 nm; the period p of a unit cell = 825 nm and the thickness t of ITO = 6 nm. The device can be fabricated using the technology of direct laser writing. The five Au-SiO_2_ stacked layer integrating the ITO film can be deposited successively using physical vapor deposition on a glass substrate, and the metagrating structure can be fabricated using femtosecond laser writing [57]. As shown in Figure 3, there are two perfect absorption peaks of over 99.9% on the curve of absorptivity (red line) at the wavelength of 1144 and 1403 nm, respectively, which means that two strong resonances occur at the two wavelengths. Next, the mechanism of perfect absorption is discussed in detail.

## 3. Results and Discussion

### 3.1. Mode Analysis

**Figure 4:**
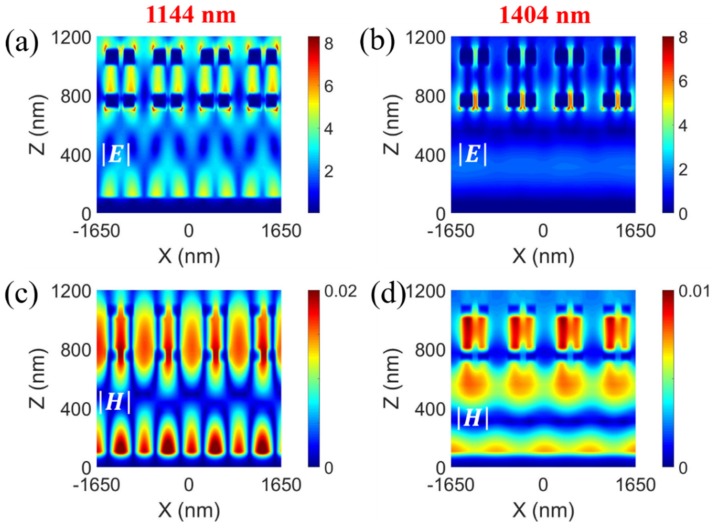
Electric field |E| and magnetic field |H| distributions at (**a**,**c**) 1144 and (**b**,**d**) 1404 nm.

**Figure 5:**
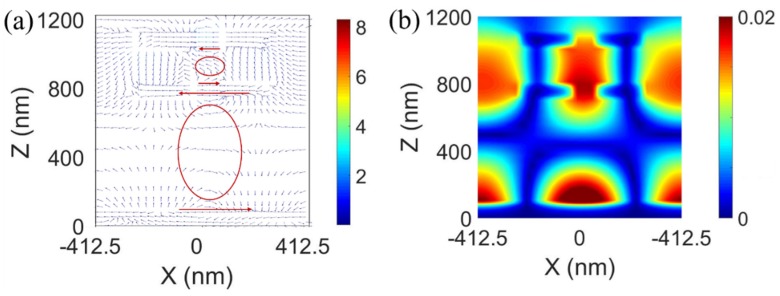
(**a**) Electric displacement |D| distribution and (**b**) magnetic field |H| distribution for one unit cell at 1144 nm.

To better understand the underlying physics of the absorption peaks at the different resonant wavelengths, the electric field |***E***| and magnetic field |***H***| distributions at 1144 (Figure 4a,c) and 1403 (Figure 4b,d) nm of the proposed structure were simulated. Figure 4a,c show that |***E***| is mainly localized in the region between the Au grating and Au mirror layer; however, |***H***| is mainly located between the region air slit and the Au mirror layer at the wavelength of 1144 nm. To determine the nature of the resonance peak, the corresponding electric displacement |***D***| and magnetic field |***H***| distributions for one unit cell are presented in Figure 5. In Figure 5a, |***D***| in the top air slit is opposite to |***D***| in the bottom air slit, which forms an anticlockwise current loop, resulting in a magnetic moment. Therefore, a strong enhancement in |***H***| was produced in the region of the air slit (Figure 5b). Antiparallel, the electric displacement was also generated in the bottom Au grating and Au mirror layer. |***H***| satisfies mirror symmetry in the top and bottom SiO_2_ regions, which means that the antisymmetric mode was excited. Therefore, the magnetic resonances induced by these two circulating currents treated as a two-cascaded circuit at 1144 nm occurred in the same direction. As a result, the magnetic field was considerably enhanced in the region of circulating currents, which enhanced the light absorption. 

On the other hand, at 1404 nm, the ENZ mode can be excited by coupling the incident light based on the metagrating structure into the 6 nm ITO film. For a continuous interface between two different media, $\varepsilon_{1}E_{1}=\varepsilon_{2}E_{2}$ (normal to the interfaces). In our case, $\varepsilon_{2}=\varepsilon_{ENZ}\ll \varepsilon_{1}$; thus, in Figure 4b, the electric field is strongly confined in the ENZ layer, enhancing the light absorption [48].

### 3.2. Electrical Modulation

**Figure 6:**
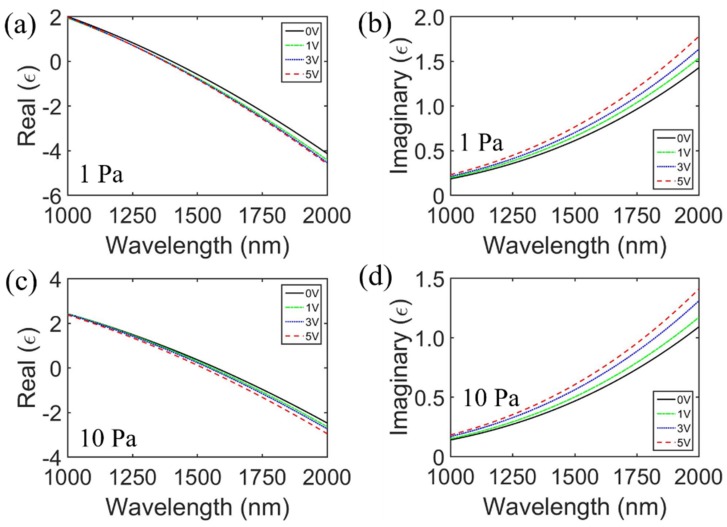
Real and imaginary permittivity of ITO films deposited under an oxygen partial pressure of 1 (**a**,**b**) and (**c**,**d**) 10 Pa for different applied voltages with wavelengths ranging from 1000 to 2000 nm.

We then addressed the modulation of the absorptivity by applying electric field gate bias. Shilin Xian et al. [56] reported that the ENZ wavelength of ITO films can be modulated under electrostatic gating and different oxygen partial pressures when the ITO films are fabricated [58,59]. The dielectric functions of ITO films deposited under an oxygen partial pressure of 1 and 10 Pa for different applied voltages were calculated using Equation (1) by fitting the experimental data from [56] and are displayed in Figure 6. 

**Figure 7:**
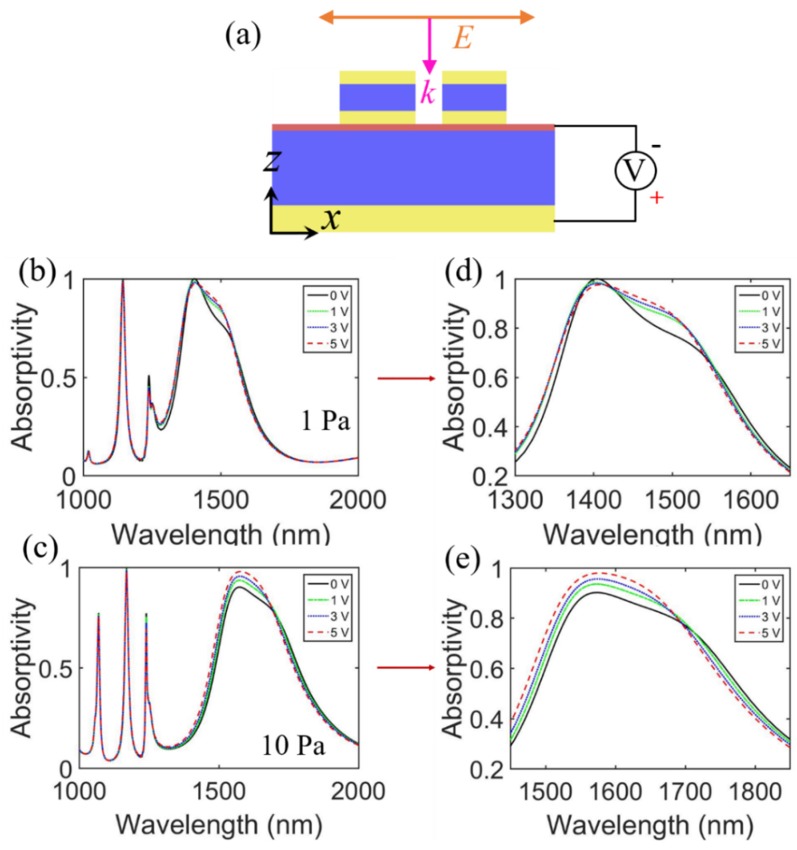
(**a**) Schematic of the MIMIM stacked structure with an applied gate voltage V. (**b**,**c**) represent the absorptivity with different applied gate voltages for ITO thin films deposited at 1 and 10 Pa. (**d**,**e**) are zoom-in views of (**b**,**c**), respectively.

To observe the modulation effects of electrostatic gating on the ENZ wavelength of ITO ultra-thin films, the calculated ENZ wavelengths of the ITO ultra-thin film under different voltages with oxygen partial pressures of 1 and 10 Pa are shown in Table 1. The ENZ wavelength decreased with an increasing voltage [56]. The simulated absorptivity under different gate voltages for ITO thin films deposited at 1 and 10 Pa is shown in Figure 7. The optimized parameters of the MIMIM structure were the same as before, except for the bottom SiO_2_ layer, with a thickness of 724 nm for ITO thin films deposited at 10 Pa. In Figure 7c, under an oxygen partial pressure of 10 Pa, it can be seen that the absorptivity near the ENZ wavelength (1570 nm) increases from 90.8% to 98.0% with the increasing applied voltage from 0 to 5 V. The absorption peak at the wavelength of 1169 nm is almost constant. Although the variation of the absorptivity for ITO fabricated at 1 Pa appears to be small, we found that the applied voltage only affects the resonant peak near the ENZ wavelength. Therefore, this characteristic can be applied to design tunable devices, such as electro-absorption modulators and reconfigurable optical biosensors.

### 3.3. Sensing Performance

**Figure 8:**
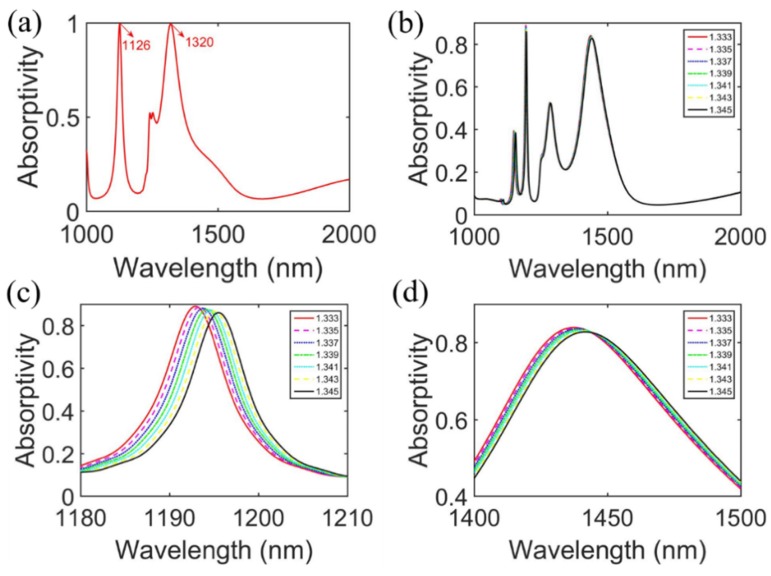
The simulated absorptivity of the optimized MIMIM structure in (**a**) air and (**b**) solutions of different refractive indices. (**c**,**d**) Refractive index-dependent absorptions of the antisymmetric mode and ENZ mode.

Finally, the sensing behaviors of the designed MIMIM structure were investigated. For most refractive index sensors based on metamaterial absorbers, the sensitivity of the sensor usually depends on the FWHM and wavelength shift per RI unit of the resonance peak. According to [8,9,18,34,60], the sensitivity of an RI sensor can be improved by reducing the FWHM of the absorber or increasing the wavelength sensitivity of the absorber to ambient RI. Figure 8a shows the calculated absorptivity of the proposed structure with the following optimization parameters: $h_{1}=h_{3}=100\;\mathrm{nm},\;h_{2}=200\;\mathrm{nm},\;h_{4}=531\;\mathrm{nm},\;l=$ 82.75 nm, p= 825 nm, and t= 6 nm. The device presents a very narrow FWHM of 8.65 nm at the resonance wavelength of 1126 nm with an absorptivity of over 99.7% and an FWHM of 97.03 nm near the ENZ wavelength.

To demonstrate that the device can be used for biological sensing, we considered a particular case: determination of the glucose solution concentration. The variation in the concentration of glucose affects the RI of the solution. Based on the previous experimental results presented in [61], the refractive index of glucose solution was obtained using the following expression:

(3) $$n=1.333+\left(25.76\times 10^{-6}\right)\times C,$$

where *C* is the concentration of glucose in millimolar units. The device was immersed in glucose solution with an RI ranging from 1.333 to 1.345 and the corresponding absorptivity with the different RI values is presented in Figure 8b. Figure 8c,d represent RI-dependent absorptions for the antisymmetric mode and ENZ mode. Figure 8c shows that the absorption shifted toward a longer wavelength with an increasing RI, while the full-width at half-maximum of the absorption peak remained basically constant at about 8.65 nm.

**Figure 9:**
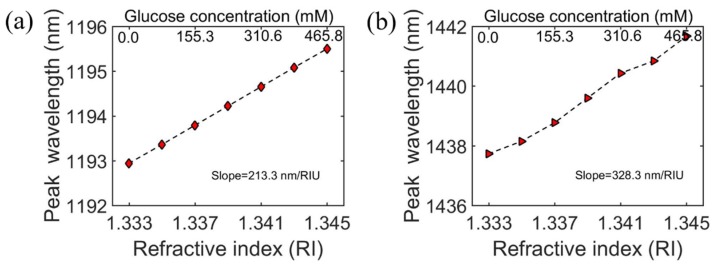
The wavelength absorption peak occurs as a function of the surrounding refractive index and exhibits different sensing performances for the (**a**) antisymmetric mode and (**b**) ENZ mode.

To obtain the sensitivity S, the peak wavelengths of absorptivity as a function of RI corresponding to the concentration of glucose for the two resonance modes were calculated and are shown in Figure 9. For the antisymmetric mode, the sensitivity S and FOM of the designed biosensor could reach 213.3 nm/RI and 24.7 in glucose solution, respectively, and 328.3 nm/RI and 3.4 for the ENZ mode. The results indicate that the proposed structure can be used as a high-performance dual-wavelength biosensor and applied in measurements of the solution concentration by detecting tiny refractive index changes.

## 4. Conclusions

In summary, we designed a dual-wavelength refractive index sensor based on a simple asymmetric metal-insulator stacked structure integrating an ENZ ultrathin film. The FWHMs of the device under normal incident light are 8.65 at the working wavelength of 1126 nm and 97.03 at 1320 nm, with over a 99.7% absorption efficiency, respectively. These two absorption peaks are generated due to excitation of the antisymmetric mode at 1126 nm and the ENZ mode at 1320 nm, based on the analysis of electromagnetic field distributions. In addition, the device can be dynamically modulated by varying the ENZ wavelength of ITO connecting to different voltages. We have demonstrated that the device can be used as a reconfigurable structure for dynamic control of the absorptivity near the ENZ wavelength. Finally, a sensing platform working within the near-infrared region was numerically proposed. In the glucose solution, the FOM of the biosensor reached 24.7 with a sensitivity of 213.3 nm/RI for the antisymmetric mode, whereas the S and FOM were 328.3 nm/RI and 3.4 for the ENZ mode, respectively. Therefore, the approach of combining the ENZ mode with a tunable wavelength and the antisymmetric mode opens new avenues for applications, including biosensors, spatial light filtering, and detectors.

## Figures and Tables

**Table 1:** The calculated epsilon-near-zero (ENZ) wavelength of the ITO ultra-thin film based on the Drude model under different voltages for ITO fabricated at 1 and 10 Pa. The left-peak value and right-peak value represent the maximum of the absorptivity at the short wavelength and long wavelength, respectively (see Figure 6b,c).

$P_{O_{2}}\;\left(\mathrm{Pa}\right)$	Voltage (V)	ENZ Wavelength	Left-Peak Value	Right-Peak Value
1 Pa	0	1403	99.8%	100%
	1	1377	99.2%	98.7%
	3	1374	99.0%	98.2%
	5	1372	98.5%	97.7%
10 Pa	0	1571	99.7%	90.2%
	1	1555	99.1%	93.5%
	3	1543	98.4%	95.6%
	5	1521	99.7%	98.0%
